# Anomalous origin of left coronary artery from pulmonary artery: A rare cause of myocardial infarction in children

**DOI:** 10.4103/1319-1683.74319

**Published:** 2010

**Authors:** Amer A. Lardhi

**Affiliations:** *Department of Pediatrics, King Fahd Hospital of the University, Alkhobar, Kingdom of Saudi Arabia*

**Keywords:** Anomalous left coronary artery arise from the pulmonary artery, anomalous origin of left coronary artery from pulmonary artery, Bland-White-Garland syndrome

## Abstract

Anomalous origin of the left coronary artery from pulmonary artery is a rare congenital heart anomaly. It presents predominantly in infancy with clinical features of myocardial ischemia and/or congestive heart failure. It poses a clinical diagnostic challenge to family physicians and pediatricians as it may present in a way similar to common pediatric conditions such as infantile colic, food intolerance, gastroesophageal reflux, and bronchiolitis. Awareness of this condition is essential for prompt diagnosis and referral to a cardiac center for early surgical intervention and improved prognosis. This article reviews this rare but serious disease in children.

## INTRODUCTION

Although the anomalous origin of the coronary arteries that arise from the pulmonary artery was first described in 1886,[1] it was not until 1933 when Bland *et al*. described the first clinical features with an autopsy finding of anomalous left coronary artery arise from the pulmonary artery (ALCAPA). The anomaly has thus been called the Bland-White-Garland Syndrome.[2,3]

Infants with the syndrome may have myocardial infarction and congestive heart failure. Without surgery, a majority of them die within the first year of life.[4] ALCAPA syndrome rarely manifests in teenagers and adults and may be an important cause of sudden cardiac arrest.[5,6] This review describes a rare but potentially lethal congenital heart disease.

## PATHOPHYSIOLOGY

**Figure 1:**
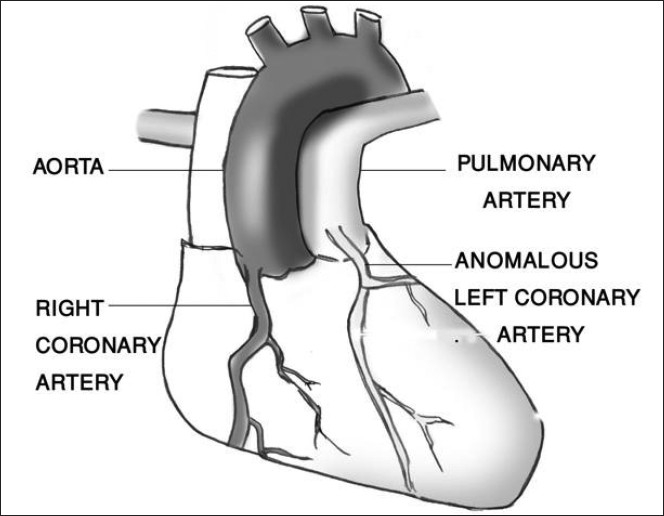
Diagram of anomalous origin of the left coronary artery from the pulmonary artery

In this anomaly, the left coronary artery arises from the pulmonary artery instead of the aorta [Figure 1]. This defect may result from either abnormal septation of the conotruncus into the aorta and pulmonary artery or from persistence of the aortic buds that eventually form the coronary arteries. The anomaly is usually isolated, but has occasionally been associated with other congenital heart defects such as patent ductus arteriosus, ventricular septal defect, tetralogy of Fallot, or coarctation of the aorta.[7,8]

In fetal life, the pulmonary artery pressure equals systemic pressure, allowing for satisfactory myocardial perfusion from the pulmonary artery through the anomalous coronary artery. However after birth, the pulmonary artery contains desaturated blood at pressure that rapidly fall below systemic pressure.[9] Therefore, the left ventricle with its huge demand for oxygen is perfused with desaturated blood at low pressure. This predisposes to myocardial ischemia, especially during exertion such as feeding or crying. Collateral vessels develop between the right and left coronary arteries and may provide adequate perfusion to the left myocardium. As the pulmonary resistance decrease further, the left coronary artery and the collateral flow tends to pass into the low-pressure pulmonary artery rather than into the high-resistance myocardial blood vessels; effectively a coronary artery steal develops from the myocardium to the pulmonary artery.[10,11] This steal phenomenon further contributes to myocardial ischemia and causes infarction of the anterolateral left ventricle free wall. The heart enlarges and congestive heart failure, which becomes manifest, is often made worse by mitral incompetence secondary to a dilated mitral ring or infarction of the papillary muscle.[12]

When collaterals are adequate, symptoms can be absent or relatively minor, allowing growth into adulthood. Adults can sometimes be asymptomatic or, more commonly, can have a variety of symptoms such as syncope, chest pain, and sudden death.[13]

### Frequency

ALCAPA is a rare congenital cardiac anomaly occurring at an incidence of 1 in 300 000 live births or 0.25 to 0.5% of all congenital heart disease.[14] However, true incidence may be greater than previously recognized, and it is possible that cases are being misdiagnosed or presenting as sudden infant death syndrome. Incidence of 1 in 4 200 to 4 800 in childhood population less than 12 years old are reported from countries in Europe.[15,16] There is no predilection for gender or race.

### Clinical features

Infants with ALCAPA appear normal at birth and usually do well for a short period before they become symptomatic, usually at 2 to 3 months (when pulmonary arterial resistance drops to adult level). Symptoms may start with a paroxysmal attack of discomfort precipitated by the exertion of nursing. This may be followed by marked pallor, irritability, and cold sweat with general appearance of shock. However, not all infants present in this way. Many present with signs and symptoms of congestive heart failure including tachypnea, tachycardia, diaphoresis, poor feeding, and poor weight gain. A few children outgrow these symptoms and gradually become asymptomatic, although periodic dyspnea, angina pectoris, or sudden death may occur in adulthood.[13,17]

### Physical examination

At physical examination, the infant may present with signs of congestive heart failure (tachypnea, rapid and weak pulse, gallop rhythm, cardiac enlargement predominantly of the left ventricle). Right ventricular enlargement, pulmonary hypertension, and loud pulmonic closure develop in long-standing left ventricle failure. If present, murmurs may be of the non specific, or the murmur of mitral incompetence. Older patients with abundant intercoronary anastomosis may have continuous murmur at the upper left sternal border.

### Diagnosis

#### Chest x-ray

**Figure 2:**
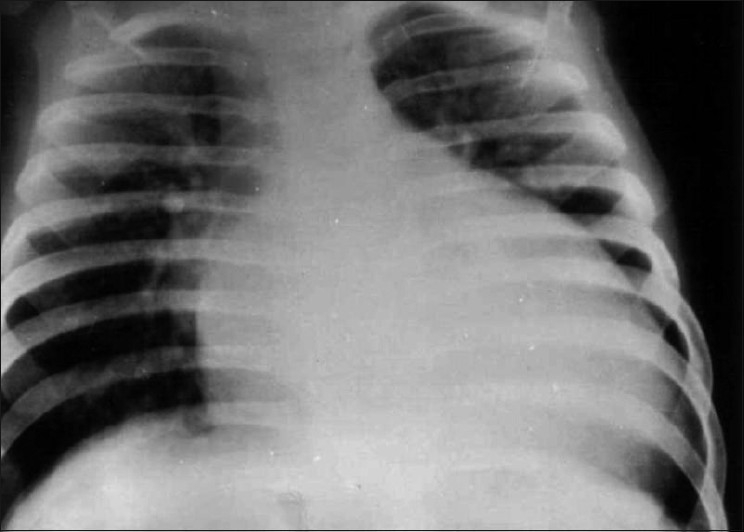
Radiographic finding of infant with ALCAPA shows cardiomegaly and pulmonary congestion

In affected infants, there is marked cardiomegaly, and evidence of pulmonary edema, a feature similar to those of many forms of cardiomyopathy [Figure 2].

**Figure 3:**
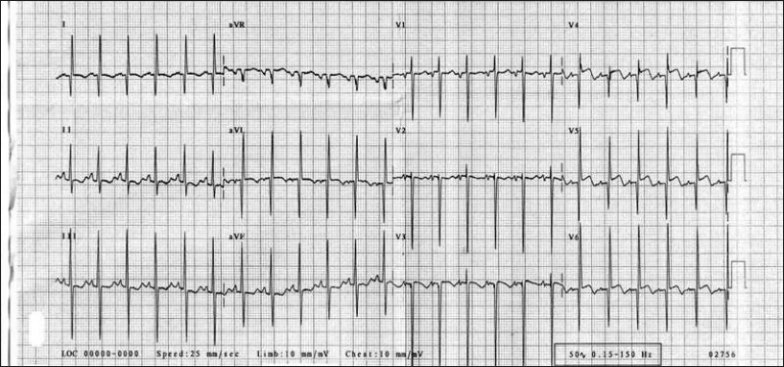
Electrocardiogram of patient with ALCAPA shows deep Q wave and inverted T wave in lead I, aVL, and left precordial leads (V5-V6)

Electrocardiogram almost always shows evidence of anterolateral myocardial infarction in the symptomatic patients [Figure 3]. A QR pattern followed by inverted T waves is seen in leads I and aVL. The left ventricular surface leads (V _5_–V _6_) may also show deep Q waves and exhibit elevated ST segments and inverted T waves.

### Echocardiography

Two-dimensional and color Doppler echocardiography is often diagnostic and, in most situations, has replaced the need for cardiac catheterization and angiography. It identifies the abnormal attachment of the left coronary artery from the pulmonary artery. Color Doppler interrogation demonstrates retrograde flow from the anomalous left coronary into the pulmonary trunk.[18] Additional imaging techniques such as computed tomography scan and magnetic resonance angiography are undertaken only when definitive diagnosis by echocardiography is not possible or in an effort to exclude other potential diagnosis.[19]

### Management

Medical management of ALCAPA consists of standard therapy for congestive heart failure that includes diuretics, afterload reduction drugs, and inotropic agents. Once the patient has stabilized, different surgical approaches have been proposed. Simple ligation of the left coronary artery at its origin from the pulmonary artery is used to prevent a steal from the myocardium.[10,20] Although often successful, it is associated with significant short- and long-term complications.[21,22] This is because after ligation, the heart is converted to one-vessel coronary system, making it entirely dependent upon right coronary artery. Current surgical procedures are directed toward establishing a two-coronary vessel system through different approaches including a direct reimplantation of the original left coronary artery in the aorta or left subclavian artery—coronary artery anastomosis, saphenous vein bypass graft, and intrapulmonary tunnel operation (Takeuchi procedure).[23–26] A significant improvement in the function of the left ventricle, mitral insufficiency, and congestive heart failure is observed after revascularization to a two-coronary artery system.

### Course and prognosis

Of all children born with this rare anomaly, about 90% present in infancy, and without surgical intervention, more than two thirds die before the age of one, usually from intractable heart failure.[4,27] A few children, may however, improve spontaneously.[28] Others with extensive collaterals may not have any symptoms. However, these patients are still at risk of sudden death due to malignant arrhythmias, especially during exercise.[23,29] Some present as adults with angina of effort or with heart failure secondary to mitral incompetence.[30]

In conclusion, ALCAPA is a rare but fatal congenital heart malformation. Family physicians and pediatricians should keep a high index of suspicion for ALCAPA during work up of any infant or child presenting with symptoms of unexplained congestive heart failure, angina-like symptoms, mitral insufficiency murmur, or cardiomegaly. Once the condition is recognized, early surgical option should be offered to the patients to avoid irreversible left ventricular dysfunction, scarring, malignant arrhythmia, and sudden death.
